# A Multispecific Checkpoint Inhibitor Nanofitin with a Fast Tumor Accumulation Property and Anti-Tumor Activity in Immune Competent Mice

**DOI:** 10.3390/biom15040471

**Published:** 2025-03-24

**Authors:** Perrine Jacquot, Javier Muñoz-Garcia, Antoine Léger, Antoine Babuty, Manon Taupin, Laurie Fradet, Fabio Dupont, Marie-Françoise Heymann, Mathieu Cinier, Dominique Heymann

**Affiliations:** 1 Affilogic SAS, 24 rue de la Rainière, 44300 Nantes, France; antoine@affilogic.com (A.L.); fabio@affilogic.com (F.D.); mathieu@affilogic.com (M.C.); 2 UMR6286, US2B, CNRS, Nantes Université, 44322 Nantes, France; javier.munoz@ico.unicancer.fr (J.M.-G.); antoine.babuty@chu-nantes.fr (A.B.); laurie.fradet@ico.unicancer.fr (L.F.); marie-francoise.heymann@ico.unicancer.fr (M.-F.H.); dominique.heymann@ico.unicancer.fr (D.H.); 3 Tumor Heterogeneity and Precision Medicine Laboratory, Institut de Cancérologie de l’Ouest, 44805 Saint-Herblain, France; 4 Nantes University Hospital, 44000 Nantes, France; 5 Research Pathology Platform, Institut de Cancérologie de l’Ouest, 44805 Saint-Herblain, France; manon.taupin@ico.unicancer.fr; 6 School of Medicine and Population Health, University of Sheffield, Sheffield S10 2RX, UK

**Keywords:** bispecific Nanofitin, programmed cell death-ligand 1 (PD-L1), epidermal growth factor receptor (EGFR), on-target/off-tumor effect, tumoral accumulation

## Abstract

Immune checkpoint inhibitors have revolutionized cancer treatment but remain limited by on-target/off-tumor effects that narrow their therapeutic window. Although PD-L1 is mainly expressed by tumor cells, these effects could reduce bloodstream availability and tumor accumulation of PD-L1 inhibitors. Enhancing tumor specificity through bispecific proteins targeting two tumor-associated antigens offers a promising strategy. This study evaluated a bispecific Nanofitin, B10–B11, targeting PD-L1 and EGFR. In vitro, B10–B11 efficiently bound to human A431 and murine CT26 cell lines, validating these models for in vivo studies. Nanofitins’ accumulation in tumors and their anti-tumor efficacy were assessed, respectively, in A431 xenograft and CT26 immunocompetent mouse models. In both experiments, B10–B11 was compared with its albumin binding fused counterpart (B10–B11-ABNF). This study showed that the dual-targeting approach with the bispecific Nanofitin enhanced in vitro PD-L1 neutralization compared to the monomeric form and led to in vivo anti-tumor activity evidenced by reduced tumor growth and increased CD3+ T cells and F4/80+ macrophages in tumors. This activity was further correlated with Nanofitin’s tumor accumulation at 7 h post-injection, which was highest for the B10–B11-ABNF. This study highlights the potential of bispecific Nanofitins, particularly with albumin binding to enable rapid and uniform tumor accumulation of effective PD-L1 immunotherapy.

## 1. Introduction

The clinical successes and Food and Drug Administration (FDA) approvals of the immune checkpoint inhibitors (ICIs) ipilimumab (Yervoy^®^, 2010), pembrolizumab (Keytruda^®^, 2016), and atezolizumab (Tecentriq^®^, 2016) targeting Cytotoxic T-Lymphocyte-Associated protein 4 (CTLA-4), Programmed cell Death protein 1 (PD-1), and programmed death-ligand 1 (PD-L1), respectively, have highlighted the relevance of addressing tumor evasion mechanisms in the management of cancer. While these therapies have fundamentally changed the paradigm of cancer treatment, their therapeutic window is restricted by on-target/off-tumor effects. These limitations hinder the ability to tailor treatment protocols to overcome the primary or acquired resistances responsible for variable efficacies across patients, tumor entities, and over time [1,2,3]. The mechanism of action of ICIs involves reactivating the immune system, which has become tolerant to tumors, which can result in Immune-Related Adverse Events (irAEs) due to uncontrolled off-tumor immune activation [4]. Anti-CTLA-4 therapy is associated with the lowest overall response rate and the highest incidence of overall and organ-specific irAEs of any grade, including grade 3 or above. This is explained by the early and systemic T-cell activation resulting from CTLA-4 neutralization, whereas the PD-1/PD-L1 pathway modulates a more restricted T-cell activation within the tumor microenvironment [5].

Nonetheless, PD-1 and PD-L1 blockers are not exempt from on-target/off-tumor effects, albeit with distinct repercussions. PD-1 neutralizing antibodies provide a general inhibition of the receptor by blocking its interaction with both programmed death-ligand 1 (PD-L1) and programmed cell death-ligand 2 (PD-L2), hence exposing to risks of pneumonitis [6]. Although PD-L1 neutralizing antibodies are more selective for the PD-1/PD-L1 axis, their systemic exposure is limited by the expression of the target protein on various non-tumoral cell types [7]. This widespread expression creates a target sink, reducing the clinical effectiveness of these antibodies. While systemic exposure can be improved by increasing the dose and saturating the non-tumoral tissues, this does not necessarily optimize the therapeutic window from a safety perspective [8]. Reducing the on-target/off-tumor effect of PD-L1 inhibitors by improving their selectivity for the tumor microenvironment could enhance their accumulation in tumors while managing safety, thus optimizing the therapeutic window.

Such selectivity improvements can be achieved by conditioning cell engagement to the recruitment of two receptors that are overexpressed in the context of malignant cells. Consequently, bispecific agents that conditionally inhibit PD-L1 only when both PD-L1 and another receptor are overexpressed on tumor cells can mitigate on-target/off-tumor effects. This approach has been described with a bispecific antibody targeting PD-L1 and Epithelial Growth Factor Receptor (EGFR) [9] and more recently by our group with a bispecific construction of the small Nanofitin alternative scaffold targeting the same two receptors [10]. In these cases, the selectivity of PD-L1 immune checkpoint blockade activity for tumor cells relies on monovalent and low-affinity interactions for each receptor, which, in the context of the co-engagement of both receptors, leads to a high-affinity interaction and subsequent immune checkpoint blockade via the so-called avidity effect.

The tumor microenvironment is characterized by elevated interstitial pressure. In this context, the distribution of macromolecules is limited by their diffusion coefficient, which decreases as their size increases [11]. Considering the high molecular weight of antibodies, they exhibit a slow and heterogeneous diffusion in tissues, which is compensated by their long plasma residence time [12]. Conversely, small alternative scaffolds like Nanofitins (7 kDa) display a high diffusion coefficient, enabling fast and efficient accumulation within the tumor tissue, despite rapid clearance from the bloodstream. This was particularly highlighted in the context of a monomeric anti-EGFR Nanofitin showing a strong and homogeneous distribution within the tumor, with a labeling of nearly all the cancer cells as fast as 90 min post-injection. Its conjugation with a cytotoxic payload provided a potent drug conjugate enabling complete inhibition of the tumor growth in a xenograft mouse tumor model [13].

In this study, we further characterized the anti-PD-L1 x EGFR bispecific Nanofitin, previously described to exhibit higher in vitro tumor cell selectivity and superior anti-tumor cell activity as compared to the monomeric anti-PD-L1 Nanofitin alone [10]. We evaluated its in vivo tumor accumulation capacity in the same tumor xenograft model as previously described for the monomeric anti-EGFR Nanofitin [13]. We also took advantage of the cross-reactivity of the anti-PD-L1 and anti-EGFR Nanofitins against the murine targets to demonstrate the in vivo anti-tumor activity of the bispecific molecule in a syngeneic immunocompetent model.

## 2. Materials and Methods

### 2.1. Plasmid Construct, Expression, and Purification of Proteins

Monomeric B10, B11 Nanofitins and albumin-binding Nanofitin (ABNF), respectively, specific to EGFR, PD-L1, and Human Serum Albumin (HSA) were used to develop multispecific Nanofitins. DNA constructs were obtained by gene synthesis (Genscript, Piscataway, NJ, USA) with codon optimization for *E. coli* expression and cloned into the pET21a vector between the NdeI and HindIII restriction sites. All final constructs were fused at the N-terminal and C-terminal with the respective hexahistidine and hemagglutinin tags. The BL21 Gold (DE3) strains were transformed by heat shock and incubated overnight at 37 °C in 10 mL of 2YT, 1% glucose, 5 ng/mL of tetracycline, and 100 ng/mL of ampicillin (growing medium) under gentle agitation (200 RPM, Innova^®^ S44i, Eppendorf, Hambourg, Germany). Then, the overnight preculture was diluted in 200 mL of growing medium and incubated at 37 °C under gentle agitation (200 RPM, Innova^®^ S44i, Eppendorf) with the regular measurement of the OD at 600 nm. When the exponential phase was reached, the protein expression was induced by adding IPTG to a final concentration of 0.5 mM (#16758-10G, Merck KGaA, Darmstadt, Germany) for 4 h at 30 °C. After protein extraction using BugBuster^®^ 1X (#70921, Merck KGaA) for 15 min, the proteins were purified by IMAC with the NGC Quest 10 Plus System (HisTrapHP, #17-5248-02, Dutscher, Bernolsheim, France) and followed by size exclusion chromatography using a HiLoad 26/600 Superdex 75 pg (#28-9893-34, Cytiva, Marlborough, MA, USA). All Nanofitins were formulated in PBS 1X 0.1M L-Arginine, 40 mg/mL Trehalose, and 0.01% Tween and filtered (Acrodisc^®^ membrane mustang^®^ E, #514-4235, VWR, Radnor, PA, USA) to remove endotoxins below 0.5 EU/mg.

### 2.2. Biolayer Interferometry Analyses

Nanofitin affinity and targeting capacity were determined by biolayer interferometry (BLI) on an Octet Red instrument (Sartorius, Goettingen, Germany). All biolayer interferometry analyses were performed in 96 multi-well plates (#655900, Dutscher) at 30 °C, 1000 RPM. The binding kinetic parameters of Nanofitins were defined by loading of 10 µg/mL mouse EGFR Fc chimera protein (#1280-ER-050, R&D Systems, Minneapolis, MN, USA) and 5 µg/mL recombinant mouse PDL1/B7-H1 Fc chimera protein (#1019-B7-100, R&D Systems) at 2 nm on protein A biosensors (#18-5012, Sartorius). All measurement steps were performed in TBS 1X containing 0.002% Tween 20 and 0.01% BSA. Between each measurement, biosensors were regenerated using three cycles of alternating washes for 10 s in Glycine 10 mM, pH 2, and TBS 1X. The biosensor unexposed to Nanofitin was used as a background reference. Sensorgrams were obtained after a reference subtraction, a background correction, a smoothing with the Savitzky–Golay algorithm and a fitting with a 1:1 model using the Octet Data Analysis software 7.1.

### 2.3. Cell Culture

All cell lines were obtained from American Type Culture Collection (ATCC) and were cultured in a 37 °C incubator with 5% CO_2_ saturated in humidity. Human A431 cell line (#CRL-1555, epidermoid carcinoma) was cultured in DMEM with 5% FBS and 1% L-glutamine, and murine CT26 cell line (#CRL-2638, colorectal carcinoma) was cultured in RPMI 1640 with 5% FBS and 1% L-glutamine. All cell lines tested negative for mycoplasma before use.

### 2.4. Cell Surface Binding by Flow Cytometry

Proliferating cells were washed with PBS and detached by 0.2% EDTA. After 5 min centrifugation at 450× *g*, the cell pellets were washed twice with cold PBS and then resuspended at 2 × 10^6^ cells/mL. Cells were distributed in 96 multi-well plates and incubated for 15 min in PBS 1X + 1% BSA. The expression levels of EGFR and PD-L1 on A431 cells were studied by incubating cells with 100 µL of the respective antibodies, PE anti-human EGFR antibody (clone AY13, 1/20, Biolegend, San Diego, CA, USA), and recombinant Alexa Fluor 488 anti-PD-L1 antibody (clone 28-8, 1/50, Abcam, Cambridge, UK). To evaluate the expression of EGFR and PD-L1 on A431 cells, the following respective control antibodies were used: PE mouse IgG1 kappa isotype control antibody (clone MOPC-21, 1/20, Biolegend) and recombinant Alexa Fluor 488 rabbit IgG (clone EPR25A, 1/500, Abcam). The expression level of EGFR on CT26 cells was studied by pre-incubating permeabilized cells with 100 µL of EGFR polyclonal antibody (#BS-0165R, 1/100, Thermo Fisher Scientific, Waltham, MA, USA) or 100 µL of the rabbit IgG isotype control (#02-6102, 1/100, Thermo Fisher Scientific) followed by incubation with the anti-rabbit IgG (H+L), F(ab’)2 fragment Alexa Fluor^®^ 488 conjugate (#4412, 1/100, Cell Signaling Technology, Danvers, MA, USA) secondary antibody. The expression level of PD-L1 on CT26 cells was studied by pre-incubating cells with 100 µL of purified anti-mouse CD274 (B7-H1,PD-L1) antibody (clone 10F.9G2, 1/50, Biolegend) or 100 µL of the purified rat IgG2b, κ isotype control antibody (clone RTK4530, 1/50, Biolegend), followed by incubation with the Alexa Fluor^®^ 488 anti-rat IgG2b (clone MRG2B-85, 1/200, Biolegend) secondary antibody. The cell binding capacity of Nanofitins was analyzed by incubating A431 cells or serum-starved CT26 cells with 100 µL of Nanofitin at 10 µM followed by the addition of DyLight650 anti-HA tag antibody (clone 16B12, 1/200, Abcam). In order to evaluate the Nanofitins’ binding capacity to cells, the control condition was performed by adding the DyLight650 anti-HA tag antibody on cells without previous incubation of Nanofitin. The FL-1 (Alexa Fluor 488), FL-2 (PE), and FL-4 (DyLight 650) fluorescence on cells were analyzed using flow cytometry (BD AccuriTM C6 Plus System, Beckman Coulter, Inc., Brea, CA, USA). For all analyses, 10,000 events were recorded.

### 2.5. Agilent X-Celligence Real-Time Cell Analysis

In total, 1 × 10^4^ A431 cells were seeded in 96 multi-well electronic plates (Agilent Technologies, Santa Clara, CA, USA). After 24 h, Nanofitin treatments were initiated by removing the medium and replacing it with fresh medium, either containing Nanofitins or not. After 1 h, a fresh medium, either containing or not containing a mix of Jurkat cells with an anti-Epithelial Cell Adhesion Molecule (EpCAM) and anti-Cluster of Differentiation 3 (CD3) Bispecific T-cell Engager (BiTE) antibody (produced by Sinobiological, Beijing, China), was added. In this experiment, A431 cells were co-cultured with Jurkat cells at a final effector/target (E:T) ratio of 10:1; Nanofitin treatments were performed at a final concentration of 10 µM; and the anti-EpCAM x CD3 BiTE was used at a final suboptimal concentration (75 ng/mL) in order to develop a background activity of Jurkat cells as previously described by Koopmans et al. [9]. The real-time kinetic proliferation of tumor cells was analyzed for 100 h with the Real-Time Cell Analysis (RTCA) system (Agilent Technologies).

### 2.6. Animal Experiments

All protocols applied in the present study were first validated by the French ethical committee of the “Pays de la Loire” (CEEA-PdL-06) and authorized by the French ministry of agriculture and fisheries (authorization APAFIS # 2168-2019080709313558 v6).

Evaluation of intra-tumoral diffusion of Nanofitins: 8-week-old female BALB/c nude mice (Janvier Labs, Le Genest-Saint-Isle, France) were anesthetized by inhalation of an isoflurane/air mixture (2%, 1 L/min) before subcutaneous inoculation of 1 × 10^6^ A431 cells in PBS into the left flank. Tumors volumes were measured every two days with a caliper according to the following formula: V = 0.5 × L × (l)^2^, in which L and l are, respectively, the largest and the smallest perpendicular diameters. On day 10 after tumor injection, homogeneous tumor sizes were obtained, and mice were randomly split into three groups of three. Single doses of B10 (66 µg), B10–B11 (131 µg), and B10–B11-ABNF (197 µg) Nanofitins were injected intravenously to achieve equal molar dose. The injected quantities of Nanofitins were determined to ensure that an equivalent quantity of anti-PD-L1 Nanofitin (around 60 µg) was distributed, as previously described [14]. Mice were sacrificed 90 min or 7 h post-injection to harvest the tumor tissues. After tumor resection and standard formalin-fixed and paraffin-embedded tissue processing, serial tumor sections were prepared for histopathological assessment.

Evaluation of Nanofitin’s effect on tumor growth: On day 0, 8-week-old female BALB/cJRj mice (Janvier Labs) were inoculated with 0.5 × 10^6^ CT26 cells in PBS by a subcutaneous injection into the left flank. On day 4, mice were randomly split into three groups of six, and Nanofitin treatments were started. The control group received intraperitoneal injection of vehicle five times a week. The B10–B11 and B10–B11-ABNF Nanofitin groups received intraperitoneal injection of 20 mg/kg Nanofitins five and three times a week, respectively, for three weeks. Starting on the sixth day after tumor cell inoculation, tumor volumes were monitored as described above, every two days. Mice were sacrificed by cervical dislocation for ethical reasons when the first critical point was detectable (e.g., high tumor size, pain, tissue necrosis). This study stopped after the sacrifice of all mice in the control group.

### 2.7. Immunohistochemistry Analyses

All immunohistochemistry (IHC) experiments were performed using BOND Polymer Refine Detection system (Leica Biosystems, Nussloch, Germany). Three-micrometer-thick sections were deparaffinized and rehydrated. Heat-induced epitope retrieval was performed using BOND Epitope Retrieval Solution 2 at pH 9 for 20 min at 95 °C (Leica Biosystems). The revelation was performed by adding a solution of an anti-rabbit polymer coupled to HRP for 8 min and then by the addition of a DAB solution. The washes were carried out in 3 cycles with Wash Solution (Leica Biosystems). Nuclei were counterstained by adding hematoxylin for 5 min. Images were acquired using a Nanozoomer digital slide scanner (Hamamatsu Photonics, Hamamatsu, Japan).

Evaluation of intra-tumoral diffusion of Nanofitins: Nanofitins were HA-tagged for tumoral detection by IHC. Following the blockade of endogenous peroxidase, tissue sections were incubated with the HA-tag rabbit mAb (clone C29F4, 1/300, Cell Signaling Technology) for 1 h.

Evaluation of Nanofitin’s effect on the immune cell recruitment: Following the blockade of endogenous peroxidase, tissue sections were incubated with the anti-CD3 epsilon antibody (ab16669, 1/150, Abcam) or the F4.80 (D2S9R) XP^®^ rabbit mAb (70076, 1/100, Cell Signaling Technology) for 15 min. CD3 and F4.80 staining percentages were estimated using Fiji-2.15.0.

## 3. Results

### 3.1. The Bispecific B10–B11 Nanofitin Binds to A431 Cells and Induces a Cytotoxic Effect

**Figure 1:**
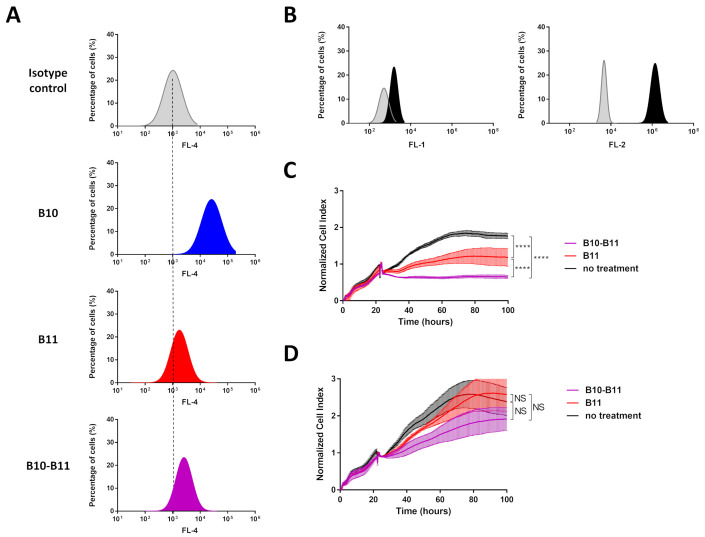
Cell labeling efficiency of the bispecific B10–B11 Nanofitin and its cytotoxic effect on the A431 on tumor cell line. (**A**) Cell labeling efficiency evaluation by flow cytometry of B10, B11, and bispecific B10–B11 Nanofitins on A431 cell line. Dotted lines represent alignment with the isotype control. (**B**) Expression level of programmed death-ligand 1 (PD-L1) (left) and Epithelial Growth Factor Receptor (EGFR) (right) on the A431 cell line. In grey: isotype control; in black: anti-PD-L1 or anti-EGFR antibody. (**C**) The real-time proliferation of A431 cells co-cultured with Jurkat cells exhibiting background activity. In black: A431 cells co-cultured with a mix of Jurkat cells and anti-EpCAM x CD3 BiTE without Nanofitin treatment; in red: A431 cells co-cultured with a mix of Jurkat cells and anti-EpCAM x CD3 BiTE and treated by the B11 Nanofitin (10 µM); in purple: A431 cells co-cultured with a mix of Jurkat cells and anti-EpCAM x CD3 BiTE and treated by the B10–B11 Nanofitin (10 µM). Significance was assessed by two-way ANOVA using 6 Prism software (GraphPad). **** *p* < 0.0001 (n = 3). (**D**) The real-time proliferation of A431 cells. In black: A431 cells alone; in red: A431 cells treated by the B11 Nanofitin (10 µM); in purple: A431 cells treated by the B10–B11 Nanofitin (10 µM). Significance was assessed by two-way ANOVA using 6 Prism software (GraphPad). NS: not significant (n = 3).

In the present study, we compared the cell labeling efficiency of the bispecific B10–B11 Nanofitin with its two monomeric counterparts on A431 cells using flow cytometry. Flow cytometry analyses revealed a stronger ability of B10 to label A431 cells than B11 (Figure 1A, Table S1). This observation was correlated with the level of EGFR and PD-L1 expression on these cells (Figure 1B). Interestingly, the bispecific B10–B11 Nanofitin demonstrated a lower cell labeling intensity than B10 but a slightly higher labeling intensity than B11 (Figure 1A, Table S1). The cell cytotoxic assay demonstrated that both B11 and bispecific B10–B11 Nanofitins induced cytotoxic effects on A431 cells cultured in the presence of Jurkat cells, with a more pronounced impact for bispecific B10–B11 (Figure 1C). In contrast, as we previously observed, the B10 monomeric Nanofitin was ineffective for inhibiting EGFR signaling in the A431 cell line [10]. Of note, bispecific B10–B11 Nanofitin also showed slight cytotoxicity in the absence of Jurkat cells through a mechanism that remains to be identified (Figure 1D).

### 3.2. Tumor Accumulation of the Bispecific B10–B11 Nanofitin in an A431 Xenograft Model

**Figure 2:**
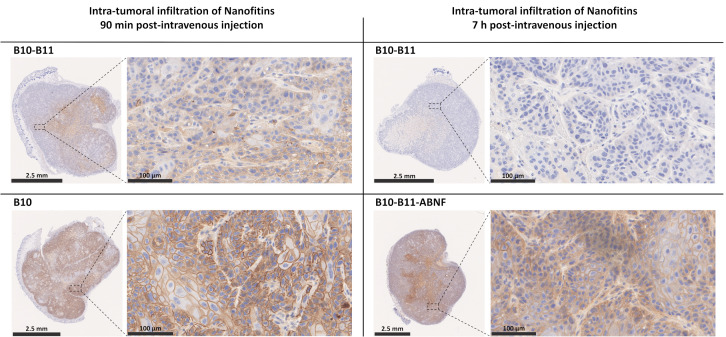
Intra-tumoral infiltration of Nanofitins after systemic administration in a A431 cell xenograft model. Intra-tumoral infiltration of B10 and bispecific B10–B11 Nanofitins were evaluated at 90 min post-intravenous injection, while bispecific B10–B11 and the bispecific Nanofitin fused to an albumin-binding Nanofitin (ABNF) were assessed at 7 h post-intravenous injection. Nanofitin staining is represented on the whole tumor (bar scale: 2.5 mm) and on a zoom of selected regions (bar scale: 100 µm). n = 3 mice per group. All tumors were analyzed, and similar results were observed across groups. Representative images are presented.

To rely on a previous report of tumor accumulation potential of the anti-EGFR Nanofitin B10 [13], tumor accumulation of the bispecific B10–B11 Nanofitin was monitored similarly in this study using the A431 tumor xenograft model. This was compared either to the monomeric B10 Nanofitin at 90 min post-injection or to the bispecific Nanofitin fused to an albumin-binding Nanofitin (ABNF) at 7 h post-injection. The localization of the different Nanofitin constructs within the tumor was revealed by immunohistochemistry on tumor tissues (Figure 2 and Figure S1). The original immunohistochemistry images can be found in Supplementary Materials (Figures S2 and S3).

The B10 Nanofitin showed both rapid tumor accumulation and diffusion, as evidenced by the positive immunostaining of the A431 cells’ membrane in nearly all the tumor tissue at 90 min post-injection. In contrast, the bispecific B10–B11 Nanofitin showed a significantly lower tumor accumulation and diffusion at the same time point, as shown by a partial staining of the tumor. At 7 h post-injection, the staining of the tumor by the bispecific B10–B11 Nanofitin remained only barely visible, suggesting that what had accumulated at 90 min post-injection had cleared by 7 h post-injection. A counterpart of this bispecific B10–B11 Nanofitin obtained by its fusion to an ABNF [15] (B10–B11-ABNF) displayed a strong accumulation and complete diffusion within the tumor tissue at 7 h post-injection.

### 3.3. The Bispecific B10–B11 Nanofitin Is Cross-Reactive with Mouse EGFR and PD-L1 and Binds to CT26 Cells

**Figure 3:**
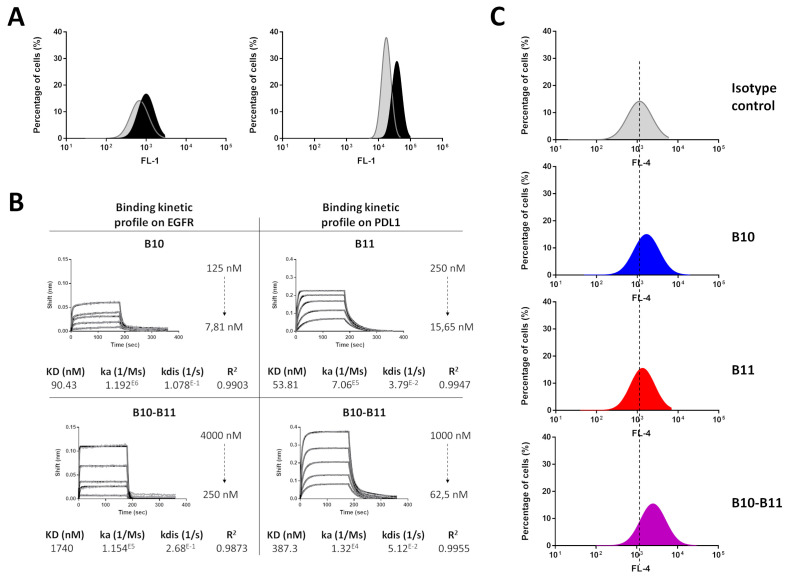
Mouse cross-reactivity of the bispecific B10–B11 Nanofitin. (**A**) Expression level of PD-L1 (left) and EGFR (right) on CT26 cell line. In grey: isotype control; in black: anti-PD-L1 or EGFR antibody. (**B**) Biolayer interferometry sensorgrams and binding kinetic parameters of monomeric B10 and B11 Nanofitins and bispecific B10–B11 Nanofitin on mouse EGFR and PD-L1. Fittings (1:1 model) are represented as solid gray lines. (**C**) CT26 cell labeling efficiency evaluation by flow cytometry of monomeric B10 and B11 and bispecific B10–B11 Nanofitins. Dotted lines represent alignment with the isotype control.

The isograft model based on inoculation of murine CT26 cells in immunocompetent mice was used to monitor an immunologic-based anti-tumor activity of the Nanofitins. Expression of EGFR and PD-L1 on the murine CT26 cell line was demonstrated by flow cytometry (Figure 3A). The cross-reactivity of the bispecific B10–B11 Nanofitin was validated by interferometry, with affinities for murine EGFR and PD-L1 of 1740 nM and 387.3 nM, respectively (Figure 3B). These affinities enable the B10–B11 Nanofitin to engage with CT26 cells at a higher level compared to its monomeric counterparts, despite the latter’s stronger affinities for EGFR (90.43 nM) and PD-L1 (53.81 nM) (Figure 3B,C, Table S1).

### 3.4. The Bispecific B10–B11 Nanofitin Reduces Tumor Proliferation in an Immunocompetent Murine Model

**Figure 4:**
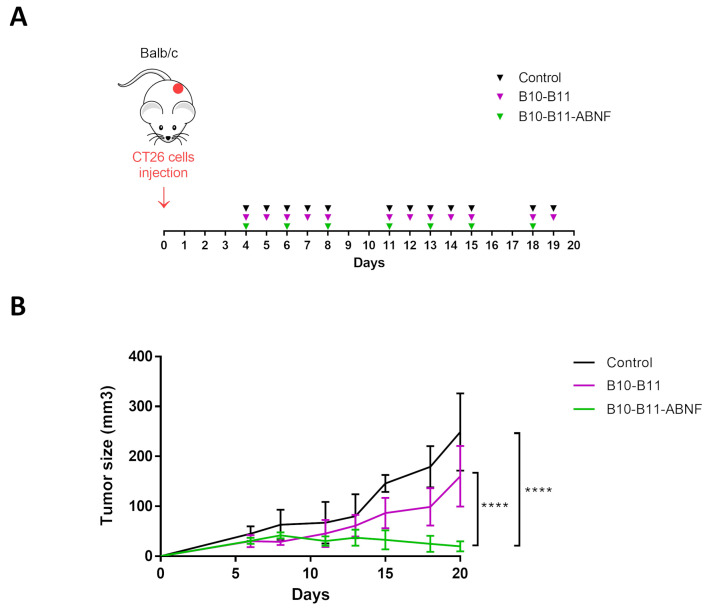
Therapeutic efficacy of the bispecific Nanofitin on an immunocompetent CT26 cell isograft model. (**A**) Study details with timeline and injection frequencies. CT26 cells (0.5 × 10^6^) were injected (s.c.) on day 0. Vehicle and B10–B11 were injected five times a week (I.P. and 20 mg/kg). B10–B11-ABNF was injected three times a week (I.P. and 20 mg/kg). Mice were sacrificed on day 20. (**B**) Tumor progression evaluation for the 3 groups of treatment. Significance was assessed by two-way ANOVA using 6 Prism software (GraphPad). **** *p* < 0.0001. Each group consisted of 6 mice (n = 6).

Immunocompetent mice bearing CT26 tumors received intraperitoneal injections starting 4 days after tumor cell inoculation when tumors were palpable in each animal. By 6 days after tumor cell inoculation, a homogeneous tumor size of approximately 40 mm^3^ was reached. Treatments involved either daily administration of the bispecific B10–B11 Nanofitin or administration every two days of the B10–B11-ABNF Nanofitin (Figure 4A). A control group received a daily injection of the vehicle only (PBS). Mouse weight was monitored throughout the experiment. Except for one mouse in the control group, during this study, no significant weight loss was observed for all the mice in the three treatment groups, suggesting that in all cases treatments were well tolerated (Figure S4). Four mice treated with the bispecific B10–B11 Nanofitin showed a partial response to the treatment. For this group, a slight reduction in tumor growth (35%) as compared to the control group was observed, though it did not reach statistical significance. However, despite less frequent administration, the treatment using the B10–B11-ABNF Nanofitin produced a much more pronounced effect. All treated mice initially showed a reduction in tumor growth, and complete inhibition of tumor growth was observed in four of them. For this group, the final tumor volume was reduced by around 92% compared to the control group (Figure 4B).

### 3.5. The Bispecific B10–B11 Nanofitin Promotes Immune Cell Recruiting

**Figure 5:**
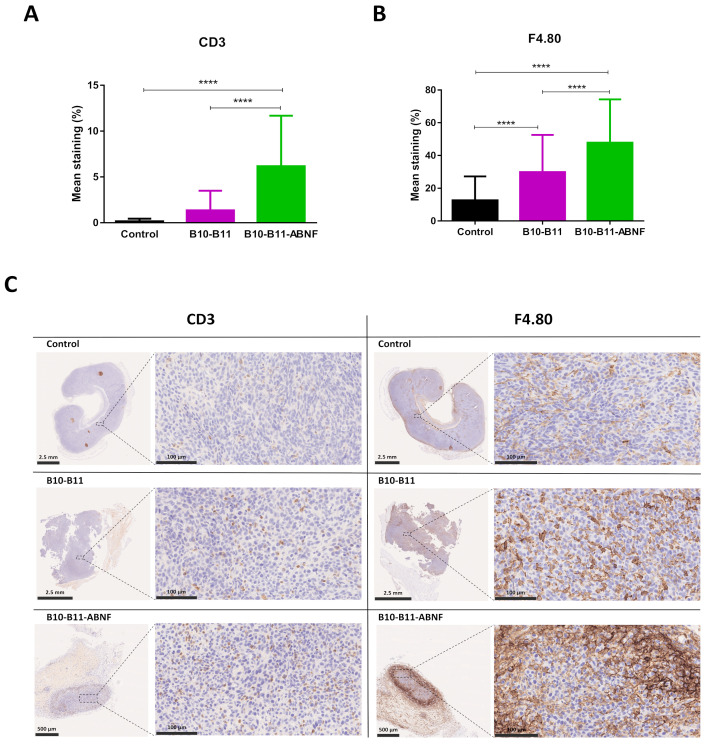
Immune cell recruitment into CT26 tumors after mice treatments. Mean staining percentages of (**A**) CD3 positive cells and (**B**) F4.80 positive cells in CT26 tumors of each treated group (control, B10–B11, and B10–B11-ABNF). Significance was assessed by one-way ANOVA using 6 Prism software (GraphPad). **** *p* < 0.0001. Each group consisted of 90 regions of interest (ROIs) measured from 3 samples per group. (**C**) CD3 and F4.80 staining of CT26 tumors for one mouse in each treated group (control, B10–B11, and B10–B11-ABNF). CD3 and F4.80 positive cell staining are represented on the whole tumor (bar scale: 2.5 mm or 500 µm) and on a zoom of selected regions (bar scale: 100 µm).

The immune infiltration of CT26 tumors was analyzed by immunohistochemistry. The original immunohistochemistry images can be found in Supplementary Materials (Figures S5 and S6). At the end point, tumor tissues were infiltrated by T cells and macrophages in treated and non-treated groups (Figure 5 and Figure S7). The semi-quantitative evaluation of these infiltrates showed a significant increase in the mean percentages of CD3+ T cells (Figure 5A,C) for the B10–B11-ABNF group and of F4.80+ macrophages (Figure 5B,C) for both treated groups compared to the non-treated group. This observation demonstrated the ability of the bispecific B10–B11 Nanofitin to recruit immune cells inside the tumor masses. Similarly, significant increases in the mean staining percentages of CD3+ T cells and F4.80+ macrophages, with respective factors of 40 and 4, revealed the superiority of the B10–B11-ABNF Nanofitin in recruiting immune cells.

## 4. Discussion

Recent progress in cancer immunotherapies has been made, but challenges remain, particularly in enhancing their therapeutic window. The high molecular weight of anti-PD-L1 antibodies, along with on-target/off-tumor uptake, limits their tumor accumulation, thus potentially compromising their effectiveness. Smaller compounds and more tumor-specific PD-L1 targeting could overcome these limitations, improving both tumor accumulation and the therapeutic window of PD-L1 inhibitors.

### 4.1. Bispecific B10–B11 Nanofitin Simultaneously Engages EGFR and PD-L1 on Human A431 and Murine CT26 Cell Lines

We previously developed a bispecific Nanofitin by fusing anti-EGFR B10 [16] and anti-PD-L1 B11 [17] Nanofitins, which demonstrated its ability to inhibit PD-L1 in an EGFR-dependent manner. Its affinities for human EGFR (892 nM) and PD-L1 (229 nM) prevented its monovalent binding to non-target cells expressing only PD-L1, even at a high level. Conversely, compared to its monovalent counterparts, the bispecific B10–B11 Nanofitin showed enhanced targeting on cells co-expressing both EGFR and PD-L1, enabling selective anti-tumor effects in vitro on MNNG-HOS tumor cells through PD-L1 neutralization, with the B10 Nanofitin having been shown to be unable to inhibit EGFR signaling [10]. In this study, we evaluated the ability of the bispecific B10–B11 Nanofitin to simultaneously bind EGFR and PD-L1 on A431 and CT26 cell lines, thereby validating these models for subsequent in vivo experiments.

First, we documented the co-expression of both EGFR and PD-L1 on A431 cells. Flow cytometry confirmed the binding of the bispecific B10–B11 Nanofitin to A431 cells. Interestingly, the cell labeling efficiency of the B10–B11 bispecific Nanofitin on A431 cells was comparable to that of the monomeric B11 Nanofitin, rather than surpassing the monomeric B10 Nanofitin. This suggests that, as previously described [10], the bispecific Nanofitin’s affinities for both human EGFR and PD-L1 prevent its monovalent binding to EGFR, as seen with the monomeric B10 Nanofitin. As a result, its binding to cells is restricted to the co-engagement of both receptors. In A431 cells, where there is a notable disparity between EGFR and PD-L1 expression levels, this leads to the labeling efficiency being aligned with that of the less-expressed receptor, i.e., PD-L1. This co-engagement likely contributes to superior in vitro immune checkpoint inhibition (ICI) activity, as seen in T-cell killing assays, compared to monomeric B11 (Figure 1). A tumor xenograft model based on A431 cells was subsequently used to evaluate the accumulation in tumors of different Nanofitin constructs.

Second, the cross-reactivity of the B10 Nanofitin on murine EGFR and the B11 Nanofitin on murine PD-L1 were confirmed with their respective affinities of 90.43 nM and 53.81 nM and their ability to target CT26 cells (Figure 3, Table S1). CT26 cells have been previously characterized by low PD-L1 expression on the cell surface (62 ± 5 receptors/cell) [18] and are yet to be sensitive to therapies based on immune checkpoint inhibition. The weak ability of the monomeric B11 Nanofitin to target CT26 cells highlights the need to enhance its PD-L1 neutralizing activity through dual targeting of EGFR and PD-L1. The affinities of the bispecific B10–B11 Nanofitin for murine EGFR (1740 nM) and PD-L1 (387.3 nM) would prevent monovalent targeting in favor of cross-arm binding, as previously described [10]. The bispecific B10–B11 Nanofitin’s superior targeting ability for CT26 cells compared to its monovalent counterparts suggest its simultaneous binding to both EGFR and PD-L1 on this murine cell line as well (Figure 3, Table S1). In vivo implantation of CT26 cells enabled the creation of an immunocompetent isograft tumor model, which was used to evaluate the therapeutic efficacy of the bispecific B10–B11 Nanofitin.

### 4.2. Nanofitin Accumulation in Tumor Is Driven by Its Size

Previously, the monomeric anti-EGFR Nanofitin B10 has shown high accumulation and broad diffusion in A431 tumor xenografts in immunodeficient mice, effectively targeting nearly all tumor cells as early as 90 min post-injection [13]. In this study, we used the same model to evaluate the in vivo tumor accumulation of the bispecific B10–B11 Nanofitin, using the monomeric B10 Nanofitin as a benchmark for fast and efficient tumor accumulation. At 90 min post-injection, the B10 monomer (~7600 Da) was widely distributed throughout the tumor while the bispecific B10–B11 Nanofitin (~16,140 Da) exhibited only partial diffusion (Figure 2). This result is consistent with other studies showing lower tumor accumulation for dimeric constructs compared to monomeric ones. Zahnd et al. observed lower tumor accumulation of the radiolabeled anti-Human Epidermal Growth Factor Receptor-2 (HER2) DARPin G3 in its dimeric form compared to its monomeric counterpart in an HER2-positive SKOV3 tumor xenograft mouse model [19]. Similarly, Debie et al. found that the monomeric anti-HER2 Nanobody 2Rb17c infiltrated tumors faster and more homogeneously than its dimeric forms made by fusion with an irrelevant Nanobody or with itself. Moreover, this homogeneity was even less pronounced for the dimeric and bivalent anti-HER2 Nanobody compared to its dimeric monovalent counterpart [20]. All of these results can be explained by a correlation between compound size and its permeability to the vasculature and diffusion within the tumor, with smaller sizes contributing more. Interestingly, although the bispecific B10–B11 Nanofitin (~16,140 Da) showed weak tumor accumulation at 90 min post-injection, compounds of similar molecular mass like DARPin (~16,900 Da) exhibited efficient tumor infiltration at the same time point. This can be explained by the non-globular nature of the B10–B11 Nanofitin, which does not follow the typical correlation between molecular weight and size observed in globular proteins. The hydrodynamic radius of the bispecific Nanofitins (~2.39 nm) is larger than that of monomeric DARPins (~1.944 nm) and similar to that of a Single-Chain Variable Fragment (ScFv) (~2.346 nm) (Table 1). In silico analysis has shown that the accumulation potential of a protein ligand as a function of its molecular size exhibits a U-shaped profile. This means there is a “Death Valley” where molecules are still too small to avoid first-pass systemic clearance and too large for efficient permeability and diffusivity, which happens to be around the size of an ScFv [21].

### 4.3. Bispecific B10–B11 Nanofitin Demonstrates a Potent Anti-PD-L1 Activity in an Immunocompetent Mice Model

The efficiency of the bispecific Nanofitin was evaluated with and without a third Nanofitin binding the Human Serum Albumin (HSA) and named ABNF. The bispecific B10–B11 Nanofitin, which has a short half-life of 10–15 min due to rapid clearance linked to its molecular weight being below the kidney’s glomerular filtration cutoff, was administered daily. The ABNF was shown to bind to serum albumin at its domain II, thereby hijacking the natural recycling mechanism of albumin to extend the plasma half-life of itself and its cargo molecule up to 20 h in mice [15]. Consequently, the B10–B11-ABNF Nanofitin was administered every other day, accounting for its longer residence in the bloodstream by the ABNF contribution.

While both constructs inhibited tumor growth, only the B10–B11-ABNF Nanofitin provided significant results, including shrinkage of the tumor by day 20 (Figure 4). Potency was correlated with increased immune cell infiltrates, particularly CD3+ T cells and F4.80+ tumor-infiltrated macrophages as quantified by IHC, compared to the vehicle control (Figure 5). The increased frequency of Tumor-Associated Macrophages (TAMs) is associated with enhanced antigen-specific T-cell activation in the CT26 syngeneic tumor model, but in a PD-L1/PD-1 interaction-dependent manner [23]. Indeed, Gordon et al. demonstrated a reduced degree of phagocytosis by PD1+ TAM compared to PD1- TAM [24]. Therefore, we hypothesize that PD-L1 blockade by the bispecific Nanofitin could release the tumor-driven hindered phagocytic activity of the TAM and thus promote antigen-specific T-cell activation. This mechanism will need to be elucidated in future studies, and it should be determined whether it could lead to anti-tumor systemic immunity by rechallenging the treated mice with a second tumor cell implantation on the other flank. Additionally, a correlation was observed between the compound efficacy in the CT26 syngeneic model and its respective tumor accumulation at 7 h post-injection in the A431 tumor xenograft model (Figure 2). It is assumed that the low tumor accumulation of the bispecific B10–B11 Nanofitin resulted from an imbalance between systemic exposure and accumulation speed, which was compensated by the incorporation of ABNF. These results suggest that the bispecific B10–B11 Nanofitin exhibits anti-tumor activity and that a minimal exposure time is necessary to trigger a significant anti-tumoral effect, which requires the addition of the ABNF.

### 4.4. Taking Advantages of Small and Bispecific PD-L1 Immune Checkpoint Inhibitor

Anti-PD-L1 antibody-based therapies face limitations in tumor delivery and efficacy due to their size and on-target/off-tumor effects. A previous study of the anti-EGFR Nanofitin showed high tumor specificity in vivo, with significant accumulation in EGFR-expressing tumors and minimal accumulation in non-EGFR-expressing tumors or healthy organs (heart, lungs, skin) 2.5 h post-injection [16]. In addition to amplifying specificity to EGFR-positive tumors, dual targeting of both EGFR and PD-L1 by a bispecific Nanofitin could address the on-target/off-tumor effects associated with PD-L1 monospecific targeting.

Additionally, smaller compounds, like the ABNF-bispecific Nanofitin, penetrate tumors faster, offering an advantage over monoclonal antibodies. This assumption aligns with Tijink et al., who observed identical tumor accumulation between cetuximab and a Nanobody trimer involving two binding sites for EGFR and one binding site for albumin, but at different timelines, as the Nanobody trimer showed faster and deeper tumor accumulation than the antibody [25]. Similarly, tumor accumulation observed as soon as 7 h post-injection for the ABNF-bispecific Nanofitin reflects that reported for monoclonal antibodies at a longer time point of at least 24 h post-injection [12].

In the present study, no weight loss or abnormal behavior was observed in mice treated with the bispecific Nanofitin, suggesting the good tolerability of the compound. Consequently, PD-L1 inhibition with the ABNF-bispecific Nanofitin, due to its small size and high tumor specificity, offers an effective strategy for bypassing both the target sink and on-target/off-tumor effects, thereby enhancing the therapeutic window of this therapy.

## 5. Conclusions

Dual targeting of two overexpressed receptors on tumor cells is a promising strategy to overcome the on-target/off-tumor effects that limit the effectiveness of PD-L1 inhibitors. Our in vitro and in vivo results show that the bispecific B10–B11 Nanofitin efficiently inhibits immune checkpoints through co-engagement of two tumor-associated antigens, EGFR and PD-L1. When fused with an albumin-binding Nanofitin, this bispecific construct generated a potent anti-tumor effect, fully inhibiting tumor growth in a CT26 syngeneic immunocompetent tumor model. Anti-tumor potency correlated with the recruitment of CD3+ T cells and F4.80+ macrophages, as well as rapid and homogeneous distribution of the Nanofitin within the tumor, observed as early as 7 h post-injection. Given its potency and selectivity, this approach could potentially expand the therapeutic window of current anti-PD-L1 therapies.

## Figures and Tables

**Table 1:** Molecular weight (kDa) versus size of scaffold and antibody-derived proteins. Molecular weight was estimated using Expasy ProtParam (Available at: https://web.expasy.org/protparam/ accessed on 30 January 2025). Stokes radius in Ångströms was estimated using HullRad (Version 10) and converted to nanometer applying the formula 1 Å = 0.1 nm. * Dimeric Nanofitin was generated by combining two structures of Sac7d (1AZP) with a 15 mer peptide linker, modeled using Modeller software (Version 9.19) [22].

Protein	PBD	Molecular Weight (kDa)	Stokes Radius (Å)	Rh (nm)
Nanofitin	1AZP	7.60	15.81	1.581
Dimeric Nanofitin	*	16.14	23.90	2.390
DARPin	5KNH	18.21	19.44	1.944
scFv	8DGR	29.59	23.46	2.346

## Data Availability

Data are contained within the article and Supplementary Materials.

## Supplementary Materials

The following supporting information can be downloaded at https://www.mdpi.com/article/10.3390/biom15040471/s1. Figure S1: A431 tumor staining by only the anti-rabbit polymer coupled to HRP. The mice were sacrificed 90 min after intravenous injection of the B10 Nanofitin. Here, the staining for one mouse of the group is represented; Figure S2: Original immunohistochemistry images of A431 tumor staining by (A) the B10–B11 Nanofitin (B) the B10 Nanofitin, 90 min post-intravenous injection; Figure S3: Original immunohistochemistry images of A431 tumor staining by (A) the B10–B11 Nanofitin (B) the B10–B11-ABNF Nanofitin, 7 h post-intravenous injection; Figure S4: Mice weight monitoring of (A) control group, (B) B10–B11 group, and (C) B10–B11-ABNF group. Measurements were taken every two days; Figure S5: Original immunohistochemistry images of CD3 positive cells staining in CT26 tumors treated with (A) Vehicle, (B) B10–B11 Nanofitin, (C) B10–B11-ABNF Nanofitin; Figure S6: Original immunohistochemistry images of F4.80 positive cells staining in CT26 tumors treated with (A) Vehicle, (B) B10–B11 Nanofitin, (C) B10–B11-ABNF Nanofitin; Figure S7: CT26 tumor staining by only the anti-rabbit polymer coupled to HRP. Data of one mouse per treated group are shown. (A) Control group, (B) B10–B11 group, (C) B10–B11-ABNF group; Table S1: Mean fluorescence intensity values of Nanofitins binding to A431 and CT26 cell lines. Comparison of mean fluorescence intensity of B10, B11, and B10–B11 Nanofitins. A DyLight650 anti-HA tag antibody was used to detect Nanofitins and as an isotype control by incubating on cells without Nanofitins. FL-4 (DyLight 650) fluorescence on cells was analyzed using flow cytometry (BD AccuriTM C6 Plus System). For each acquisition, fluorescence measurements were conducted on a total of 10,000 recorded events.

## Abbreviations

The following abbreviations are used in this manuscript:

FDA	Food and Drug Administration
ICIs	Immune checkpoint inhibitors
CTLA-4	Cytotoxic T-Lymphocyte-Associated protein 4
PD-1	Programmed cell Death protein 1
PD-L1	Programmed death-ligand 1
irAEs	Immune-Related Adverse Events
PD-L2	Programmed cell death-ligand 2
EGFR	Epithelial Growth Factor Receptor
ABNF	Albumin-binding Nanofitin
HSA	Human Serum Albumin
EpCAM	Epithelial Cell Adhesion Molecule
CD3	Cluster of Differentiation 3
BiTE	Bispecific T-cell Engager
RTCA	Real-Time Cell Analysis
IHC	Immunohistochemistry
scFv	Single-Chain Variable Fragment
TAMs	Tumor-Associated Macrophages
