# Supplementary material for: A Multispecific Checkpoint Inhibitor Nanofitin with a Fast Tumor Accumulation Property and Anti-Tumor Activity in Immune Competent Mice
Source: Biomolecules. 2025 Mar 24;15(4):471. doi: 10.3390/biom15040471 (PMC12024894; doi:10.3390/biom15040471)
Supplement: Supplementary file 1 [file biomolecules-15-00471-s001.zip › Supplementary figures.pdf]

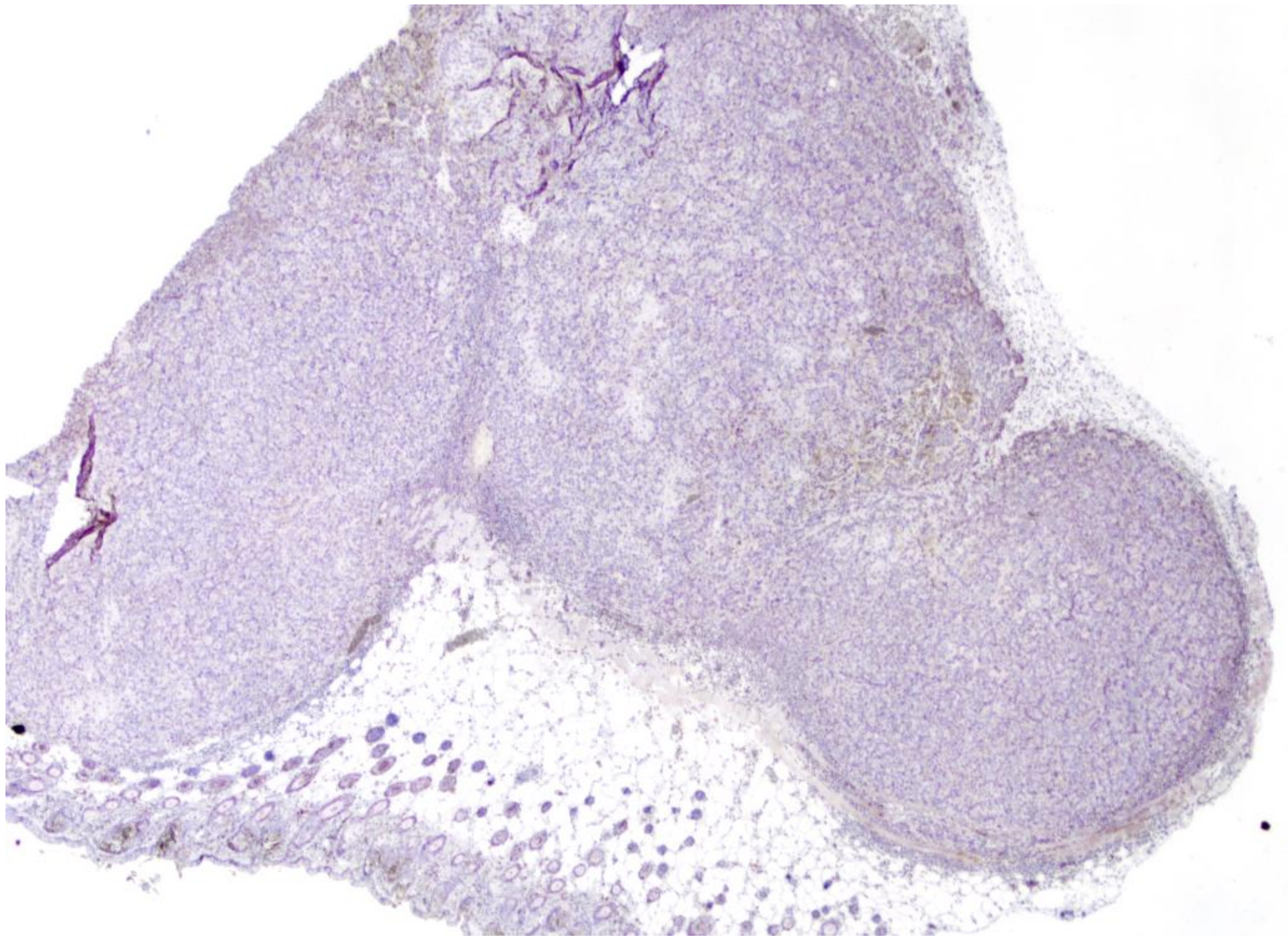

Figure S1: A431 tumor staining by only the anti-rabbit polymer coupled to HRP. The mice were sacrificed 90 min after intravenous injection of the B10 Nanofitin. Here, the staining for one mouse of the group is represented.

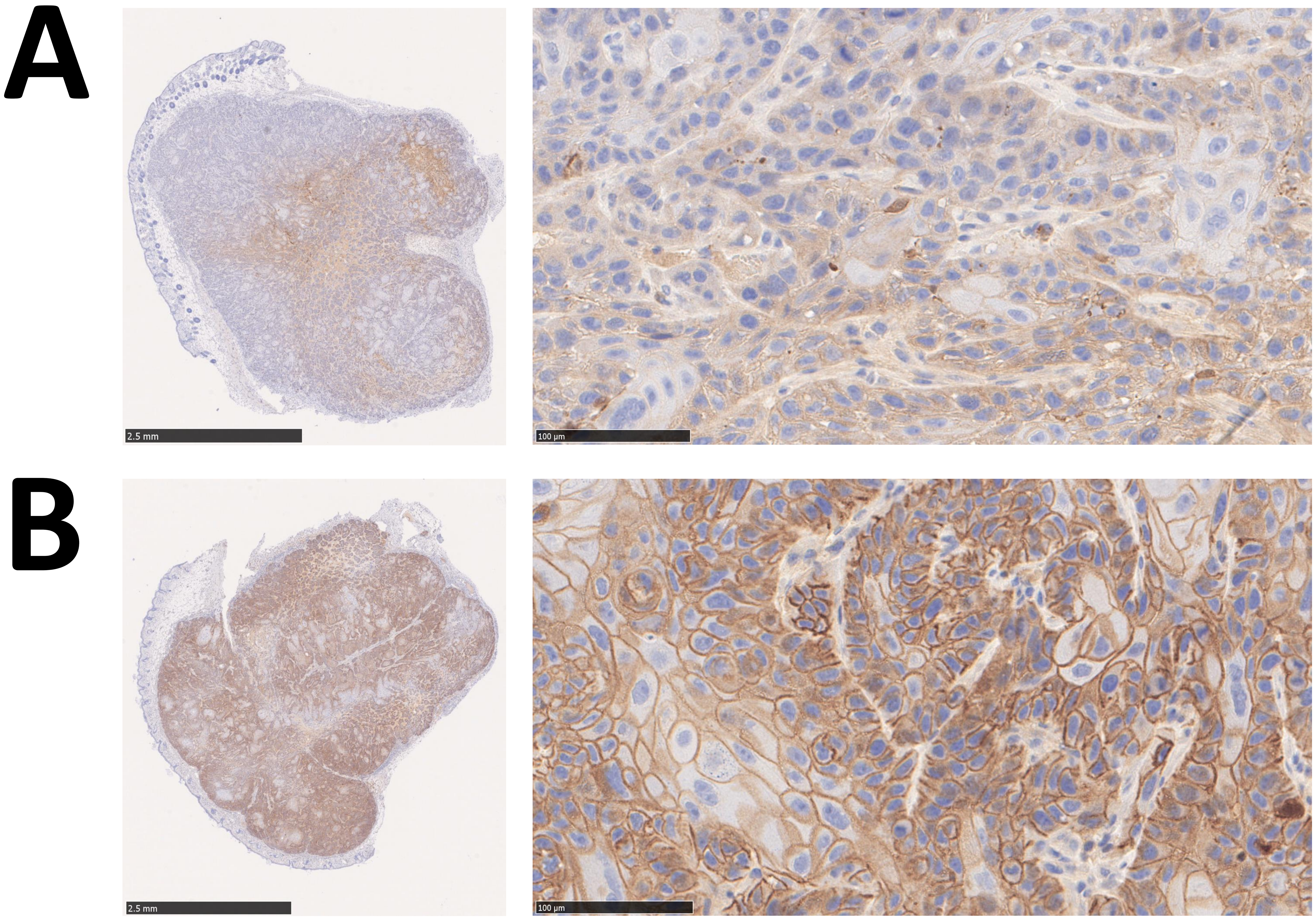

Figure S2: Original immunohistochemistry images of A431 tumor staining by (A) the B10–B11 Nanofitin (B) the B10 Nanofitin, 90 min post-intravenous injection.

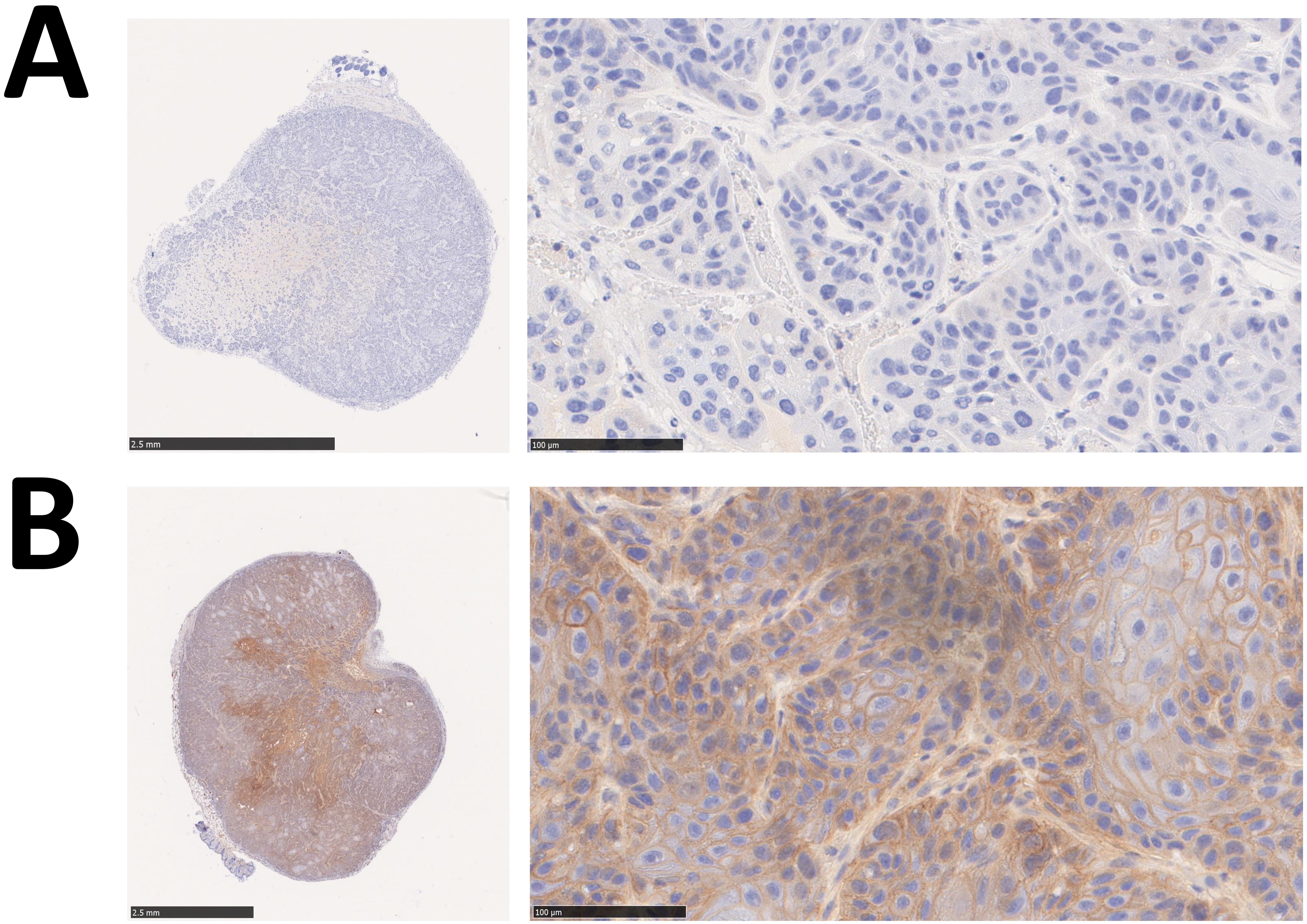

Figure S3: Original immunohistochemistry images of A431 tumor staining by (A) the B10–B11 Nanofitin (B) the B10–B11-ABNF Nanofitin, 7 h post-intravenous injection.

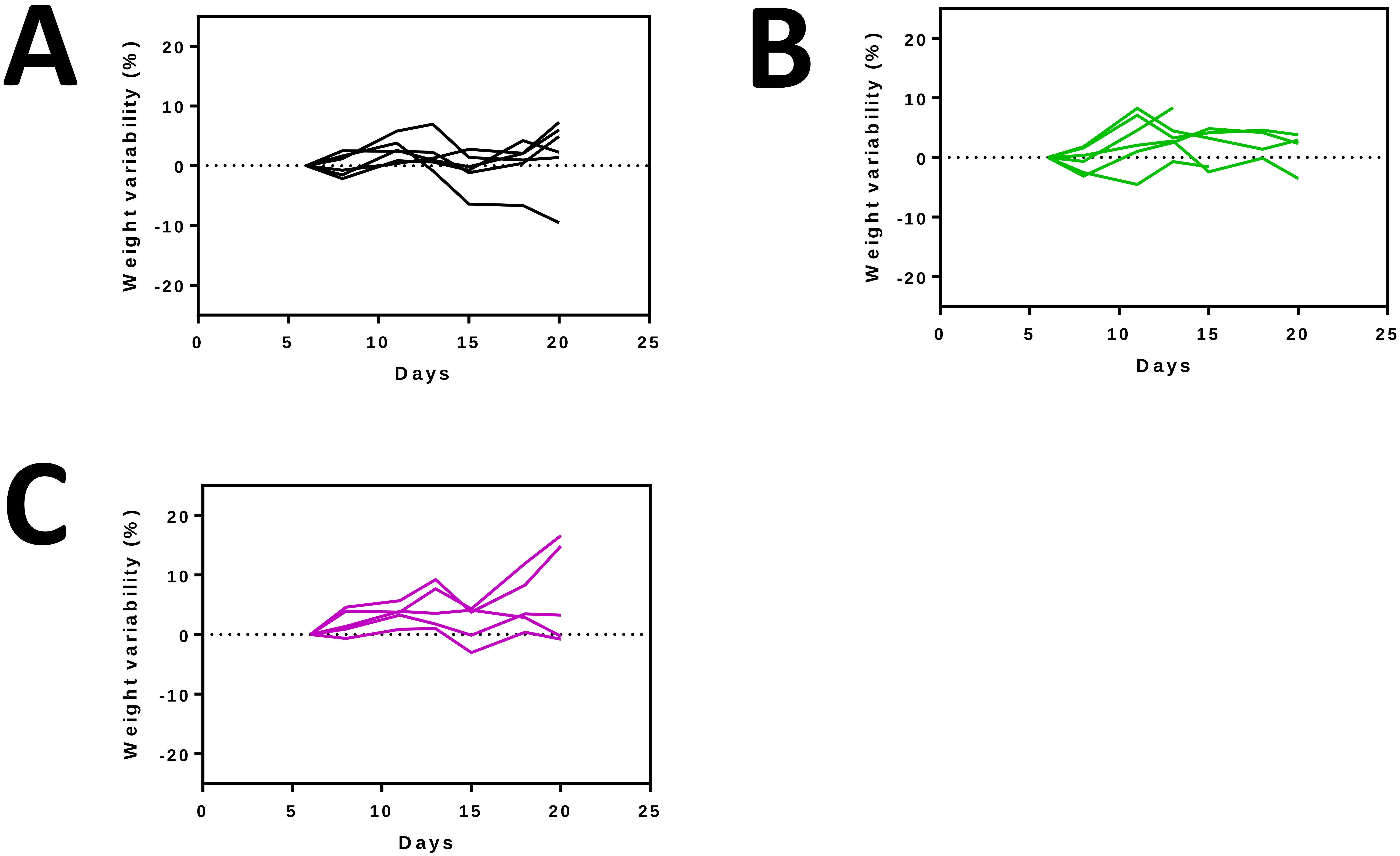

Figure S4: Mice weight monitoring of (A) Control group, (B) B10–B11 group and (C) B10–B11-ABNF group. Measurements were taken every two days.

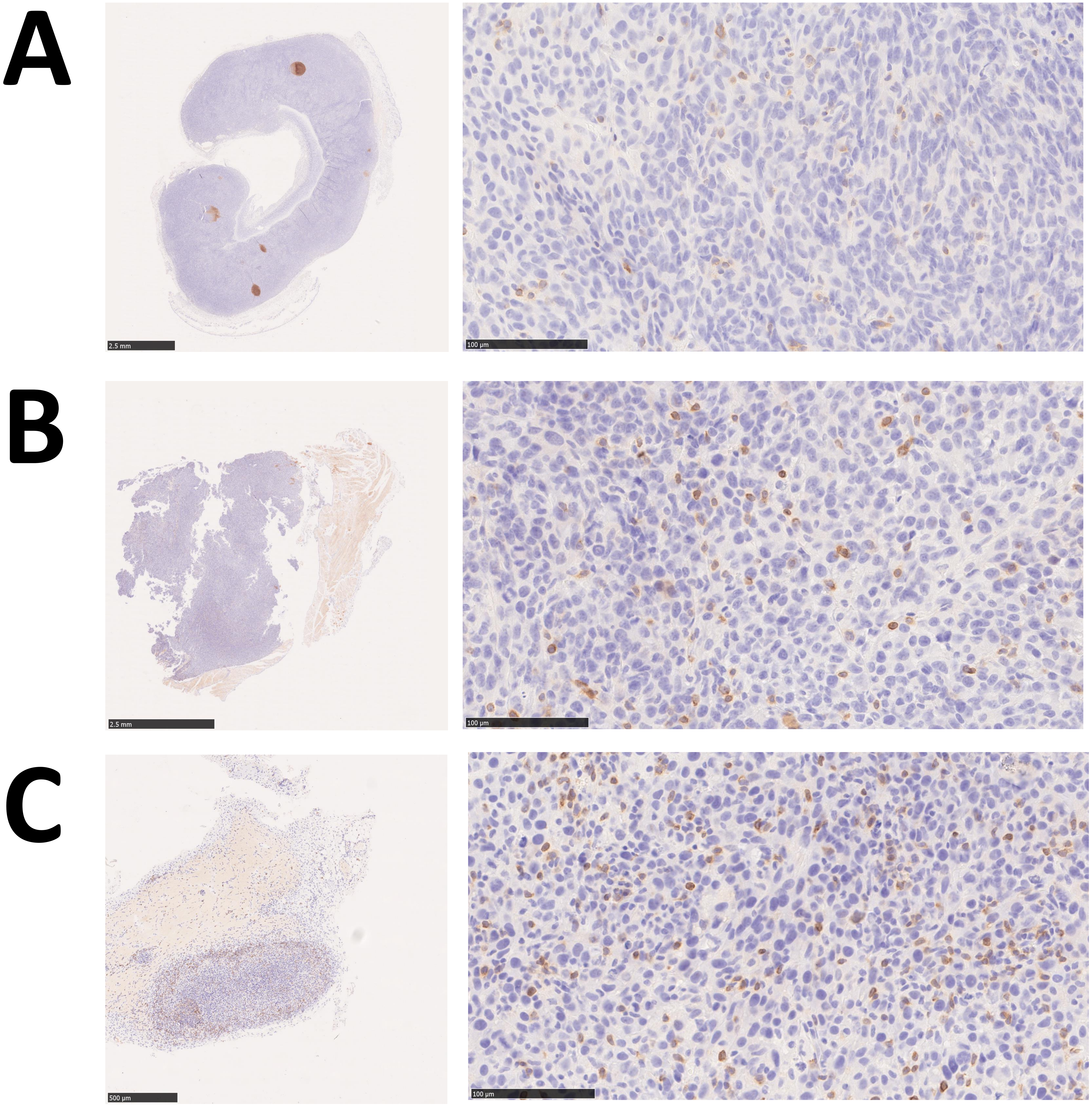

Figure S5: Original immunohistochemistry images of CD3 positive cells staining in CT26 tumors treated with (A) Vehicle, (B) B10–B11 Nanofitin, (C) B10–B11-ABNF Nanofitin.

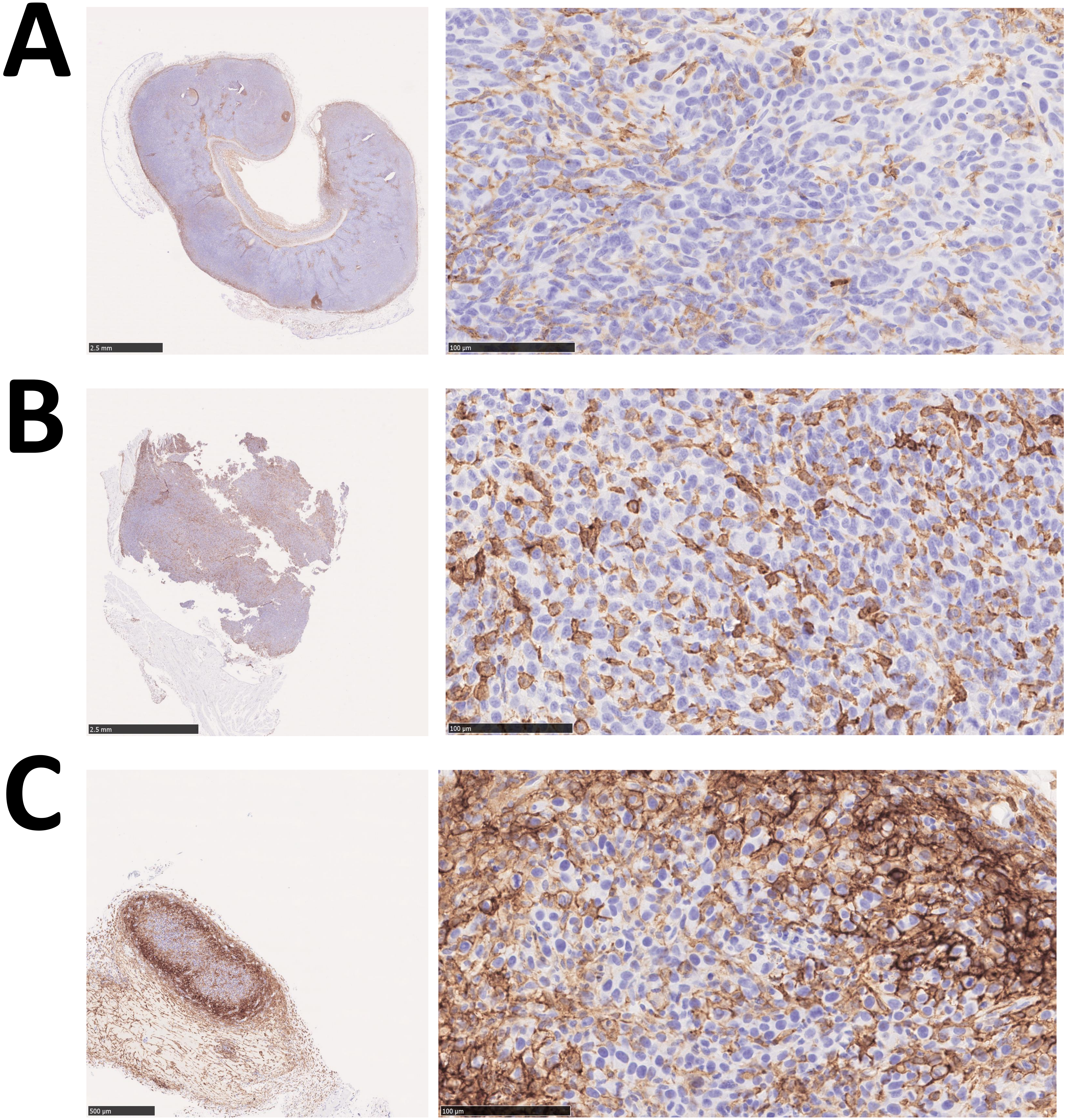

Figure S6: Original immunohistochemistry images of F4.80 positive cells staining in CT26 tumors treated with (A) Vehicle, (B) B10–B11 Nanofitin, (C) B10–B11-ABNF Nanofitin.

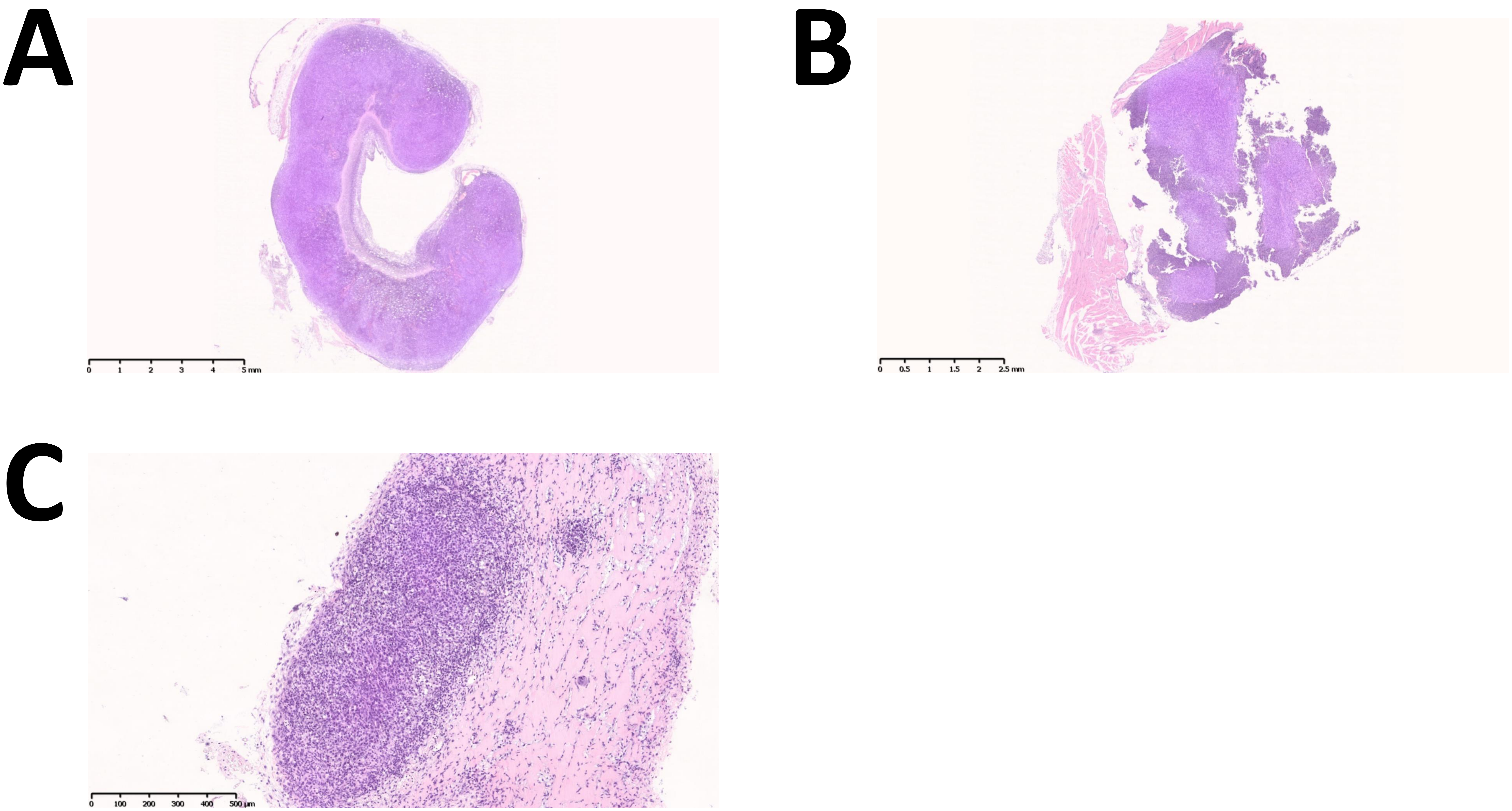

Figure S7: CT26 tumors staining by only the anti-rabbit polymer coupled to HRP. Data of one mouse per treated group are shown. (A) Control group, (B) B10-B11 group, (C) B10-B11-ABNF group.
