# Supplementary material for: A Multispecific Checkpoint Inhibitor Nanofitin with a Fast Tumor Accumulation Property and Anti-Tumor Activity in Immune Competent Mice
Source: Biomolecules. 2025 Mar 24;15(4):471. doi: 10.3390/biom15040471 (PMC12024894; doi:10.3390/biom15040471)
Supplement: Supplementary file 1 [file biomolecules-15-00471-s001.zip › Supplementary tables.pdf]

Cell line	Condition	Mean Fluorescence Intensity
**A431**	Cells only	880.3
	Isotype control	1012.72
	B10	17469.6
	B11	1214.8
	B10-B11	2811.9
**CT26**	Cells only	730.79
	Isotype control	835.99
	B10	3719.09
	B11	1204.01
	B10-B11	4460.83

Table S1: Mean fluorescence intensity values of Nanofitins binding to A431 and CT26 cell lines. Comparison of mean fluorescence intensity of B10, B11, and B10–B11 Nanofitins. A DyLight650 anti-HA tag antibody was used to detect Nanofitins and as an isotype control by incubating on cells without Nanofitins. FL-4 (DyLight 650) fluorescence on cells was analyzed using flow cytometry (BD AccuriTM C6 Plus System). For each acquisition, fluorescence measurements were conducted on a total of 10,000 recorded events.
